# Case report: severe reversible cardiomyopathy associated with systemic inflammatory response syndrome in the setting of diabetic hyperosmolar hyperglycemic non-ketotic syndrome

**DOI:** 10.1186/s12872-015-0112-3

**Published:** 2015-10-14

**Authors:** Justin Berk, Raymond Wade, Hatice Duygu Baser, Joaquin Lado

**Affiliations:** Department of Internal Medicine, Texas Tech University Health Sciences Center, School of Medicine, 3601 4th St Stop 9410, Lubbock, TX 79416 USA

**Keywords:** Cardiomyopathy, Diabetes, Systemic inflammatory response syndrome, Non-thyroidal illness syndrome, Hyperosmolar hyperglycemic non-ketotic syndrome

## Abstract

**Background:**

This case study features a woman who presented with clinical and laboratory findings consistent with hyperosmolar hyperglycemic non-ketotic syndrome (HHNS), systemic inflammatory response syndrome (SIRS), and non-thyroidal illness syndrome (NTIS) who was noted to have a transient decrease in myocardial function. To our knowledge, this is the first case discussing the overlapping pathophysiological mechanisms could increase susceptibility to SIRS-induced cardiomyopathy. It is imperative that this clinical question be investigated further as such a relationship may have significant clinical implications for prevention and future treatments, particularly in patients similar to the one presented in this clinical case.

**Case presentation:**

A 53-year old Caucasian female presented to the Emergency Department for cough, nausea, vomiting and “feeling sick for 3 weeks.” Labs were indicative of diabetic ketoacidosis. Initial electrocardiograms were suggestive of possible myocardial infarction and follow-up echocardiogram showed severely depressed left ventricular systolic function which resolved upon treatment of ketoacidosis.

**Conclusion:**

We suggest that her cardiomyopathy could have three synergistic sources: SIRS, HHNS and NTIS. Overlapping mechanisms suggest uncontrolled diabetes mellitus and NTIS could increase susceptibility to SIRS-induced cardiomyopathy as seen in this case. HHNS and SIRS cause cardiac tissue injury through mechanisms including impairment of fatty acid oxidation and formation of reactive oxygen species, as well as modifying the function of membrane calcium channels. As a result, it is conceivable that diabetes may amplify the deleterious effects of inflammatory stressors on cardiac myocytes. This novel case report offers a path for future research into prevention and treatment of SIRS-induced cardiomyopathy in, but not exclusive to, the setting of diabetes.

## Background

This case study features a woman who presented with clinical and laboratory findings consistent with hyperosmolar hyperglycemic non-ketotic syndrome (HHNS), systemic inflammatory response syndrome (SIRS), and non-thyroidal illness syndrome (NTIS) who was noted to have a transient decrease in myocardial function. We suggest that her cardiomyopathy could have three synergistic sources: SIRS, HHNS and NTIS. Overlapping mechanisms suggest uncontrolled diabetes mellitus and NTIS could increase susceptibility to SIRS-induced cardiomyopathy as seen in this case.

## Case presentation

A 53-year old female presented to the Emergency Department for cough, nausea, vomiting and “feeling sick for 3 weeks.” She reported an allergy to penicillin but no other significant past medical history. On initial assessment, patient was afebrile, tachycardic (125 beats/minute), tachypneic (22 breaths/minute), with blood pressure of 109/74 mmHg, and oxygen saturation of 72 % on room air. Physical exam showed no other abnormalities.

**Fig. 1:**
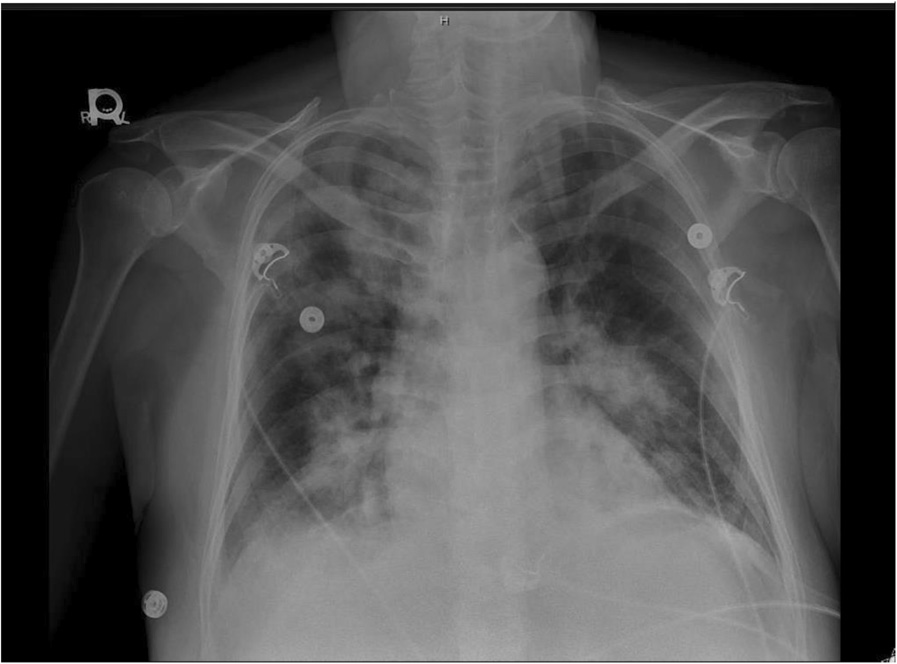
Chest X-ray, Portable film notable for bilateral, patchy hilar infiltrates consistent with pulmonary edema in the setting of depressed ejection fraction

Initial laboratories showed leukocytosis (WBC 22,000 k/mcgl), hyperglycemia (glucose 796 mg/dl), hyponatremia (Na 120 mEq/L; corrected 131 mEq/L), a hemoglobin A1c of 17.2 %, and an elevated troponin T (0.19 ng/mL) and BNP (2137 pg/mL). Measured osmolarity was 349 mOsm/L with only small ketones in the blood and an arterial lactate of 5.62 mmol/L. Arterial blood gas suggested metabolic acidosis (pH = 7.264 | PCO2 = 25.9 mmHg | Bicarbonate = 13 mmol/L). Calculated anion gap was 30 mEq/l. Chest X-ray showed bilateral reticular opacities without cardiomegaly (Fig. 1). The patient was also found to have an abnormal thyroid hormone profile suggesting NTIS: TSH 1.35 mcgUI/mL (nr: 0.27–4.20), free T4 0.88 ng/dL (nr: 0.93–1.70), free T3 0.95 pg/ml (nr:2.30–4.20), mixed hyperlipidemia, mild elevation of lipase and amylase, and transaminitis.

**Fig. 2:**
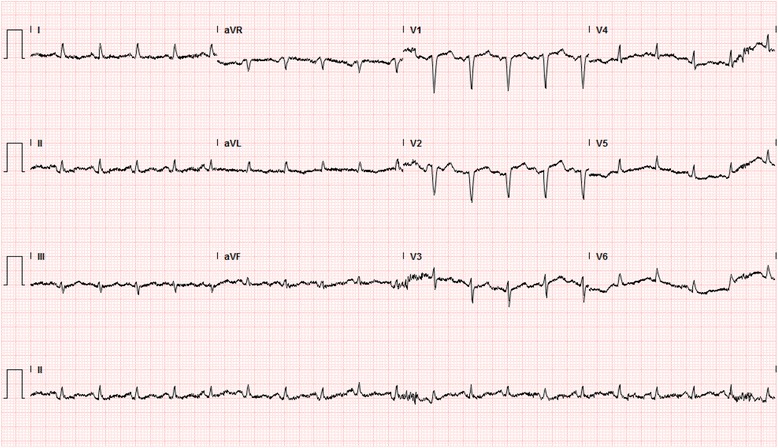
Initial EKG, EKG taken at admission notable for q-waves in the septal leads consistent with prior myocardial infarction. There are no findings concerning for acute ischemia

**Fig. 3:**
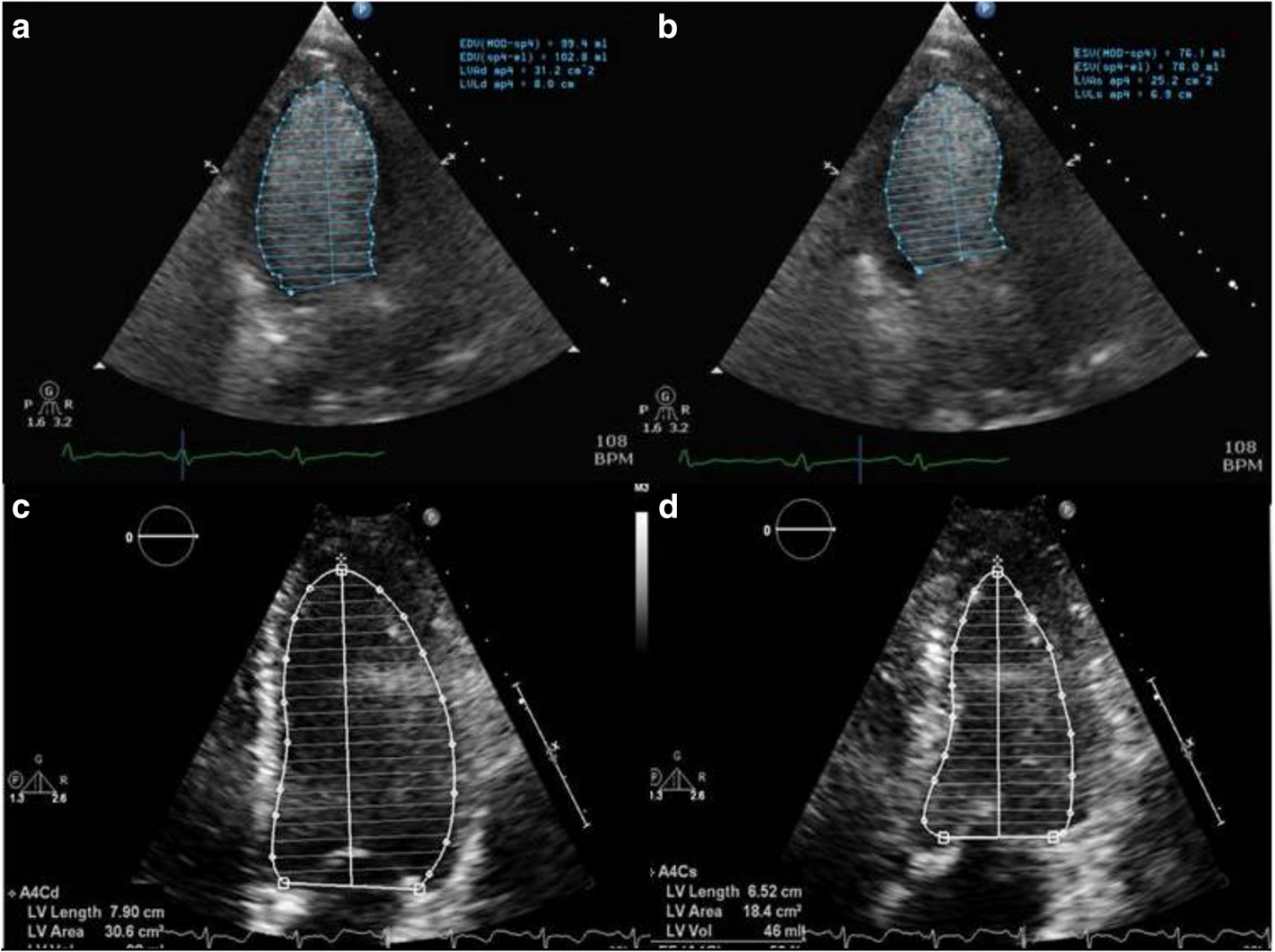
Transthoracic echocardiogram at Day 1 and Day 7, Apical four chamber view from day 1 transthoracic echocardiogram (TTE) with IV contrast at end-diastole **a** and end-systole **b** demonstrates depressed left ventricular (LV) ejection fraction estimated at 26 %. Apical four chamber view from day 7 TTE at end-diastole **c** and end-systole **d** demonstrates normalized LV ejection fraction

**Fig. 4:**
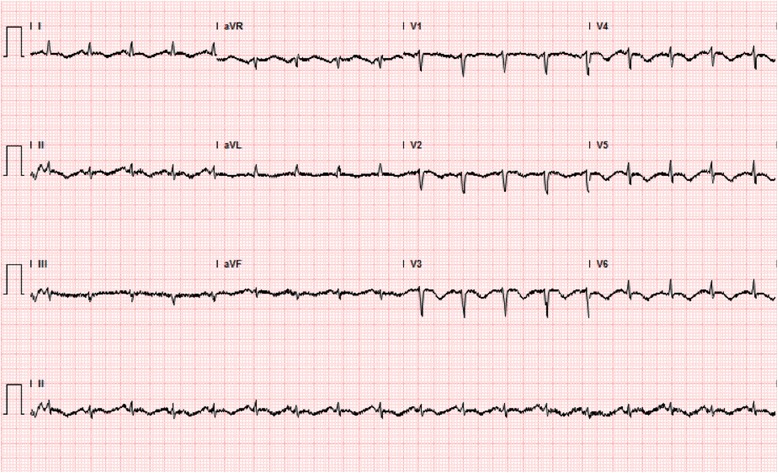
Follow-up EKG on Day 4 of hospitalization

Intravenous fluids, insulin and levofloxacin were started; the patient’s clinical condition improved in 24 hours, although she continued to complain of shortness of breath and remained hypotensive. Blood and urine cultures were ultimately negative. Initial EKG changes suggested previous myocardial infarction (Fig. 2). A transthoracic echocardiogram demonstrated a severely depressed LV systolic function (ejection fraction = 26 %), grade 2 diastolic dysfunction, and multiple regional wall motion abnormalities (Fig. 3a-3b). However, on Day 4 after admission, a Myoview stress test showed no stress-induced ischemia, normal LV function, no regional wall motion abnormalities, and an estimated ejection fraction of 50 %. A repeat transthoracic echocardiogram performed on Day 7 demonstrated an ejection fraction of 50–55 % (Fig. 3c-3d). A follow-up EKG on Day 4 showed no significant changes (Fig. 4).

On 6-month follow-up, the patient’s A1c remained elevated but the NTIS and cardiomyopathy had resolved. Her HgA1c was 9.4 %, TSH 1.86 mcgUI/mL (nr: 0.27–4.20), free T4 1.01 ng/dl (nr: 0.93–1.70), free T3 2.89 pg/ml (nr:2.30–4.20). BNP was 42 pg/ml (normal).

## Discussion

Our patient presented a case of severe reversible myocardial dysfunction with HHNS as debut of diabetes mellitus, NTIS, and meeting SIRS criteria. Although sepsis of pulmonary origin was initially suspected, the clinical course (normal body temperature, normalization in blood leukocytes count in less than 24 and negative cultures) suggest that SIRS was secondary to HHNS [1]. The observed NTIS was also likely secondary to HHNS and SIRS.

Myocardial dysfunction is a common complication in patients with SIRS secondary to sepsis and is associated with an increased risk of mortality of up to 70–90 % [2, 3]. Systolic and diastolic myocardial dysfunction has been described in other situations of SIRS such as severe trauma and burns [4].

A common mechanism among these clinical situations is a high level of pro-inflammatory cytokines such as tumor necrosis alpha (TNF-alpha) and interleukin 6 (IL-6). Bacterial products and pro-inflammatory cytokines increase production of nitric oxide [5–7] and reactive oxygen species (ROS) [8, 9] that inhibit the function of proteins involved in myocardial contraction and relaxation [9, 10].

The sarcoendoplasmic reticulum adenosine triphosphatase 2a (SERCA2a) and sarcolemmal voltage-gated L-type calcium channels are often affected. As a consequence, Ca^2+^ entry into cells and release from sarcoplasmic reticulum (SR) decreases and sarcomere shortening is reduced [11, 12]. Bacterial products and pro-inflammatory cytokines also inhibit *SERCA2a* gene expression [13], reduce myofilament sensitivity to Ca^2+^ [14], and downregulate and desensitize beta-adrenergic receptors [15, 16].

SIRS can cause myocardial energy deficiency**.** Low ATP levels and a decrease in the phosphocreatine/ATP ratio have been found in deadly cases of septic shock [17–19]. Both glucose oxidation and fatty acid oxidation (FAO), the main sources of myocardial ATP, decrease in non-surviving humans with septic shock [20]. Pyruvate dehydrogenase complex [21] and phosphofructokinase activities [22] decrease during septic shock leading to dissociation of glycolysis from glucose oxidation.

Factors responsible for FAO inhibition during septic shock are a decrease in mitochondrial membrane carnitine shuttle activity and a decrease in peroxisome proliferator-activated receptor alpha and peroxisome proliferator-activated receptor gamma co-activator-1α gene expression [23, 24]. Also, mitochondrial complexes I, II and III activities decrease in cases of prolonged septic shock [25, 26]. Multiple reviews have been published to discuss these and other possible mechanisms of sepsis-induced cardiomyopathy [8, 27–30].

Acute hyperglycemic crisis can cause SIRS, and both hyperosmolar hyperglycemic non-ketotic syndrome and diabetic ketoacidosis are associated with a severe inflammatory state [31]. However, other features of hyperglycemic crisis, such as dehydration and electrolyte imbalance, likely exacerbated the patient’s cardiomyopathy.

Diabetic cardiomyopathy refers to ventricular dysfunction in the absence of coronary artery disease and hypertension [32]. Diastolic and systolic dysfunction can be seen as early functional alteration of diabetic cardiomyopathy [32]. The pathogenic mechanisms involved are complex, interrelated and not all well characterized, although inflammation seems to play a significant role [32]. Under normal circumstances, the heart obtains energy mostly from FAO. In diabetic hearts, glucose uptake, glycolysis and glucose oxidation are reduced [32] and FA uptake increased. An increase in FA uptake is associated with increased triglycerides synthesis that causes myocardial lipotoxicity and cell death. Also, higher FAO rates increase oxygen consumption at the expense of mitochondrial uncoupling and increased oxidative stress. Our patient had elevated troponin T levels indicating cellular death or myocardial cell membrane alterations. Additionally, the hyperosmolar state may cause dehydration, which would decrease preload and further lower left ventricular systolic function.

Our patient also presented with low thyroid hormone levels with normal TSH, a condition known as NTIS or sick euthyroid syndrome. Although NTIS is probably the most common cause of hypothyroidism, the current consensus advises against the administration of thyroid hormones to patients with NTIS as thyroid hormones increase respiratory rate, oxygen consumption, energy expenditure and heat production, NTIS is considered an adaptive response to counteract catabolism during illness [33], and this is the primary reasoning against administration of thyroid hormone to patients with NTIS [34]. However, SIRS is associated with myocardial hypothyroidism in large animal models of septic shock [35] and hypothyroidism can cause systolic and diastolic myocardial dysfunction. In this context, thyroid hormone administration, in attempt to normalize thyroid hormone levels inside the myocardium, could have been beneficial and devoid of harmful effects. In fact, hypothyroidism increases serum levels of troponin T and creatine kinase CK-MB isoenzymes that are markers of myocardial damage. Patients with heart failure and low serum T3 have poor hemodynamics and a higher probability of death [36]. Finally, thyroid hormone administration to patients with advanced congestive heart failure was well tolerated and increased cardiac output without an appreciable increase in ischemia or arrhythmias [36–38].

## Conclusion

As we have explained, both HHNS and SIRS cause cardiac tissue injury through similar mechanisms including impairment of fatty acid oxidation and formation of reactive oxygen species, as well as modifying the function of membrane calcium channels. As a result, it is conceivable that diabetes may amplify the deleterious effects of inflammatory stressors on cardiac myocytes. It is imperative that this clinical question be investigated further as such a relationship may have significant clinical implications for prevention and future treatments, particularly in patients similar to the one presented in this clinical case.

## Consent

Written informed consent was obtained from the patient for publication of this Case Report and any accompanying images through standard institutional protocol. A copy of the written consent is available for review by the Editor-in-Chief of this journal.

## Abbreviations

SIRS: Systemic Inflammatory Response Syndrome
HHNS: Hyperosmolar Hyperglycemic Non-Ketotic Syndrome
NTIS: Non-thyroidal Illness Syndrome
FAO: Fatty Acid Oxidation
